# The Prescription Pattern of Chinese Herbal Products That Contain *Dang-Qui* and Risk of Endometrial Cancer among Tamoxifen-Treated Female Breast Cancer Survivors in Taiwan: A Population-Based Study

**DOI:** 10.1371/journal.pone.0113887

**Published:** 2014-12-08

**Authors:** Chien-Tung Wu, Jung-Nien Lai, Yueh-Ting Tsai

**Affiliations:** 1 Institute of Traditional Medicine, School of Medicine, National Yang-Ming University, Taipei, Taiwan; 2 Department of Chinese Medicine, Taipei City Hospital, Yangming Branch, Taipei, Taiwan; 3 Taiwan Association for Traditional Chinese Medicine of Family, Taipei, Taiwan; The Institute of Cancer Research, United Kingdom

## Abstract

**Purpose:**

The increased practice of traditional Chinese medicine worldwide has raised concerns regarding herb-drug interactions. We analyzed the usage of Chinese herbal products containing *dang-qui* and investigated whether *dang-qui* therapy increases endometrial cancer risk among tamoxifen-treated breast cancer survivors in Taiwan.

**Methods:**

All patients newly diagnosed with invasive breast cancer who received tamoxifen treatment from January 1, 1998, to December 31, 2008 were selected from the National Health Insurance Research Database. The usage, frequency of service and type of Chinese herbal products containing *dang-qui* prescribed across the 31,970 survivors were evaluated. Logistic regression method was employed to estimate the odds ratios for utilization of Chinese herbal products containing *dang-qui*. Cox proportional hazard regression was performed to calculate the hazard ratio of endometrial cancer associated with *dang-qui* use within the cohort.

**Results:**

Almost one in two study subjects had used *dang-qui*. Among 31,938 tamoxifen-treated breast cancer survivors, 157 cases of subsequent endometrial cancer were identified. The hazard ratio for development of endometrial cancer among breast cancer survivors aged 20–79 years who had taken *dang-qui* after tamoxifen treatment was decreased compared to survivors who had never used *dang-qui* (HR: 0.61, 95%CI: 0.44–0.84). To minimise potential confounding factors, women with breast cancer in the reproductive age were excluded from further analysis, and the negative relationship between *dang-qui* consumption and subsequent endometrial cancer among breast cancer survivors aged 55–79 years was still observed, although not significantly (HR: 0.74, 95%CI: 0.46–1.17).

**Conclusions:**

*Dang-qui* consumption is common among breast cancer survivors aged 20–79 years and seems decrease the risk of subsequent endometrial cancer after less than a cumulative dose of 7,500 mg of tamoxifen treatment.

## Introduction

Tamoxifen is a nonsteroidal triphenylethylene derivative that is currently used for the treatment for ER+ (estrogen receptor positive) breast cancer as a means of reducing recurrent breast cancer in breast cancer survivors (BC survivors) [1]–[6]. However, previous reports have indicated that there is an increased risk of second primary cancers in the endometrium associated with longer tamoxifen treatment and this risk needs to be considered clinically when treating postmenopausal BC survivors with tamoxifen and for at least 5 years after the last treatment [7], [8]. In addition, tamoxifen can induce depression, hot flushes, and/or uterine abnormalities [9] and these involve a complex interaction between cancer-related symptoms and cancer-related mood disorders [10]. As a result of the above factors, many women with breast cancer often use complementary and alternative medicines (CAMs), including herbs, vitamins, homeopathic remedies and Chinese herbal products (CHPs), to help relieve these symptoms [11], [12].

*Dang-qui* (*Angelica sinensis radix*) has a long history of use as part of the traditional Chinese pharmacopoeia since it was documented in the classical Chinese text *Ben Cao Gang Mu* (Compendium of Materia Medica) circa 1,590 A.D. by Shi-Zhen Li [13]. In the classical literature, *dang-qui* is said to nourish the blood and calm the nerves such that it eventually reduces gynecological disorders, menopausal symptoms, fatigue, and anemia. *Dang-qui* is often used in combination with other herbs to form various carefully balanced prescriptions that are tailored to a range of different gynecological ailments by traditional medicine practitioners. Japanese, South Korean, Chinese, and Taiwanese traditional medicine practitioners have a similar history of traditional Chinese medicine (TCM) and when prescribing Chinese herbal products (CHPs) rely on the patient's indications and contraindications, as mentioned in the thousand-year-old classical literature of Chinese medicine, for their guidance. Among the top ten most frequently prescribed Chinese herbal product (CHP) for treating breast cancer in Taiwan, seven contain *dang qui* [14]. Despite its popularity, *dang-qui* does not have any scientific and clinical evidence to back up claims of efficacy or safety among BC survivors who are receiving tamoxifen treatment.

Individuals in Taiwan are free to choose from care offered by western medicine clinics or by TCM clinics. With a health insured rate over 98% of the whole population of Taiwan, the National Health Insurance Research Database (NHIRD) is representative of the general population of Taiwan and should allow a reasonably accurate assessment of the co-utilization of TCM and modern medical resources in Taiwan. Therefore, the NHIRD provides an ideal platform for pharmaco-epidemiological studies [15]–[17]. Our study's aim was to describe the demographics and patterns of usage of CHP containing *dang-qui* medicines among tamoxifen-treated BC survivors from a Taiwanese nationwide cohort and to explore the subsequent endometrial-cancer risk among these individuals. Our findings provide evidence-based information that will help the formulation of appropriate management strategies in terms of consumption of *dang-qui* and exploring the potential risk of endometrial cancer due to an interaction *dang-qui* and tamoxifen. Obviously, integrating both technologies should be beneficial to the overall health of BC survivors and result in a strengthening of the patient-physician relationship during breast cancer care.

## Methods

### Data resources and study sample

Our study protocols were approved by the Institutional Review Board of the Taipei City Hospital. It was designed as a population-based study to determine the prevalence of CHP containing *dang-qui* (CHP-*dang-qui*) use among tamoxifen-treated BC survivors between January 1, 1998, and December 31, 2008 and to investigate the association between having been prescribed CHP-*dang-qui* and the occurrence of subsequent endometrial cancer in Taiwan. All data were obtained from the reimbursement database maintained by national health insurance (NHI). In March of 1995, Taiwan established the NHI programme, which routinely reimburses more than 96% of Taiwanese residents for the cost of prescribed medicines since 1997. Taiwan's NHI is a government-run, single-payer national health insurance scheme, financed on a pay-as-you-go basis through a mix of premiums and taxes. Therefore, all beneficiaries are designed to designate where the assets will go when they visit hospital or clinics in person or before they died. The electronic records of the NHIRD used in this study have encrypted identification numbers for all beneficiaries and the dataset was transformed and is maintained by the National Health Research Institutes of Taiwan [18], [19]. The NHIRD records contain demographic information including age and sex, as well as clinical data, which includes all records of clinical visits and hospitalizations together with all information regarding prescribed drugs and dosages, including those for CHPs and single herbs such as *dang-qui*. The diagnoses used in the NHIRD are coded according to the International Classification of Diseases, Ninth Revision, Clinical Modification (ICD-9-CM) [20].

**Figure 1:**
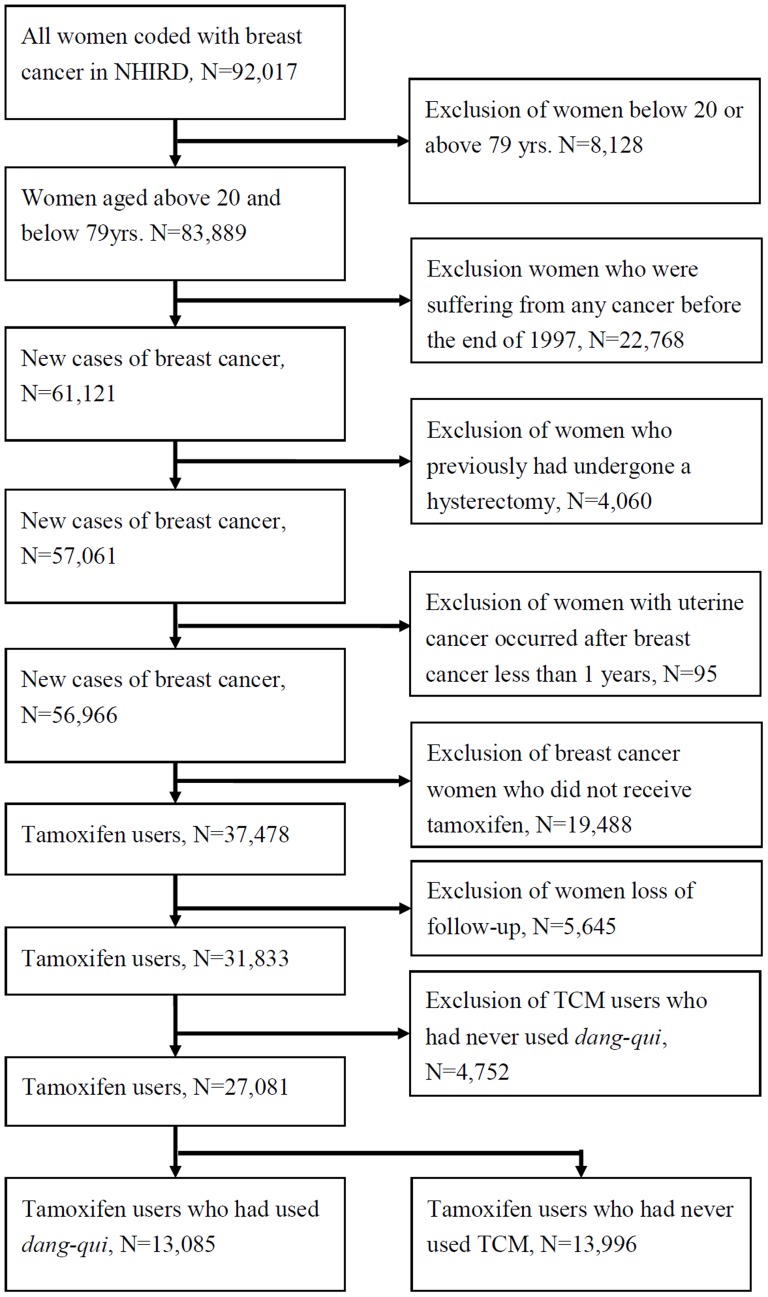
Algorithm explaining the recruitment of subjects from the National Health Insurance catastrophic illnesses registry of Taiwan for the years 1998 to 2008.

To select potential case subjects for this study, we first obtained the NHI catastrophic illness registry files for all patients who were diagnosed with breast cancer from January 1, 1998, to December 31, 2008. All patients who were confirmed to have a serious illness in these files are exempt from all copayments and therefore the presence of invasive breast cancer (ICD-9: 174) must have been microscopically confirmed to qualify for the registry; as a result this dataset is very comprehensive. The selection of study subjects was performed as follows Fig. 1. First, women who were diagnosed with breast cancer before 20 or over 79 years of age (n = 8,128) were excluded to limit the study sample to adult cancer patients in Taiwan. Second, we excluded prevalent cases of any cancers (ICD-9: 140–239, including breast cancer) diagnosed before the end of 1997 to make sure that all the subjects included were newly diagnosed with invasive breast cancer (BC survivors) during 1998–2008 (n = 22,768). Furthermore, to control for various potential confounding factors, we further excluded BC survivors who undergone a hysterectomy (n = 4,060), or whose diagnosis of uterine cancer occurred less than one year after their breast cancer (n = 95), and who did not receive tamoxifen (n = 19,488) to make sure that all the subjects included were tamoxifen-treated BC survivors. We identified all BC survivors 20 years of age or older who were continuously enrolled in the NHIRD during the years 1998 to 2008. We recorded the starting and stopping date, and dosage for each period of tamoxifen treatment. Entry to the analysis was defined as the date at which a tamoxifen-treated BC survivor first received a prescription for tamoxifen; this date was also used to calculate her age at diagnosis of endometrial cancer. Again, to study the prescription pattern and effect of *dang-qui* or/and Chinese herbal formulas containing *dang-qui*, we excluded TCM users who had never used *dang-qui* (n = 5,540). Finally, 31,938 tamoxifen-treated BC survivors (non TCM users, n = 17,004; *dang-qui* users, n = 14,934) were included in the study cohort. Because the NHI system has a comprehensive coverage for TCM prescriptions, the cost of which is generally less than the cost of herbs sold in Taiwan's markets, the likelihood that non TCM users which might serve as a reference group purchased and consumed *dang-qui* outside the NHI database is not high.

### Traditional Chinese medicine

A wide range of TCM treatments, including CHP, acupuncture and manipulative therapies for trauma, are all reimbursed by the NHI of Taiwan. CHPs consist of one or more herbs (the formula) and are the most widely adopted TCM treatment used by patients in Taiwan [21]. To study the utilization of prescribed CHP containing *dang-qui* in the present study, we downloaded the detailed herbal contents of all reimbursed CHPs from the website of the Department of Chinese Medicine and Pharmacy, Ministry of Health and Welfare, Taiwan (DCMP); the information included the name of each herb, the proportion of each constituent in the mixture, the date and period of approval for the drug, the manufacturer code, and the name of the CHP manufacturer [19].

### Study variables

To determine the key independent variables for the utilization of CHP containing *dang-qui* among tamoxifen-treated BC survivors, we selected a number of demographic factors that had been used by previous studies [14]. Age was defined as the year of tamoxifen consumption minus the year of birth and categorized into six groups: 20–29, 30–39, 40–49, 50–59, 60–69, and 70–79 years. The geographic areas of Taiwan in which patients resided were classified into one of the following five regions: Northern region, Central region, Eastern region, Southern region and Outlying islands. We split the monthly income of the subjects into four levels: New Taiwan Dollars (NT$) 0, 1–19,999, 20,000–39,999, and ≥40,000. The cumulative dose and defined daily dose (DDD) of tamoxifen were defined as the sum usage of each record in the database began from the index date and ended at the endometrial cancer diagnosis, the death of patient, withdrawal from Taiwan's Health Insurance program, or the end of 2008, whichever occurred first. And the cumulative dose of tamoxifen administered in the same period was categorized into four groups: <7,500 mg, 7,500–14,999 mg, 15,000–29,999 mg, ≥30,000 mg. According to the document of the WHO Collaborating Centre for Drug Statistics Methodology, 20 mg is the assumed average maintenance dose per day DDD for tamoxifen used for its main indication in women with hormone receptor-positive breast cancer. The cumulative DDD of tamoxifen administered were categorized into four groups: <180 DDD, 180–599 DDD, 600–1,199 DDD, ≥1,200 DDD. Cancer treatment modalities were categorized into four groups: tamoxifen-alone, tamoxifen plus surgery, tamoxifen plus chemotherapy, and tamoxifen plus surgery plus chemotherapy. Under the restriction of Taiwan's NHI, the medical record of all tamoxifen-treated BC survivors leaving Taiwan who will be not able to visit clinics in person or died from any diseases before either the diagnosis of endometrial cancer or the end of the study in which condition health service fee will be no longer covered by NHI will be not reachable in the present study. Furthermore, we also analyzed the risk of subsequent endometrial cancer between *dang-qui* users and non TCM users.

### Statistical analysis

Data are presented using descriptive statistics for the prescription rates of CHP-*dang-qui* users and are stratified by patient's age, indications for the prescription of CHP-*dang-qui*, and the most frequently prescribed herbal formulae. Potential predictors (covariates) were risk factors which may increase the chance of using *dang-qui* (demographic factors: age at diagnosis, insured salaries, insured regions; cumulative doses of tamoxifen, and cancer treatment modalities). The following 3 models corresponding to three possible underlying biological situations regarding the *dang-qui* use across multiple conditions were tested: model 1, adjusted for the demographic factors (age, insured salaries, insured region); model 2, adjusted for the demographic factors, cumulative doses of tamoxifen, cancer treatment modalities, and risk factors (diabetes mellitus, hypertension); model 3, adjusted for the demographic factors, cumulative doses of tamoxifen, and cancer treatment modalities. The value of the model was tested with the likelihood ratio test. It can be assessed by comparing the fit of the two models with and without the independent variables. Overall fit of model 3 shows a strong relationship between most of the independent variables, taken together, and dependent variable.

Primary indications were classified according to the ICD-9. The diagnoses were coded according to the ICD-9 and grouped into various broad disease categories. For example, ICD-9 codes 460-519 were classified as diseases of the respiratory system; codes 780–799 were grouped as symptoms, signs, and ill-defined conditions, and codes 520–579 were classified as diseases of the digestive system. Multiple logistic regression was conducted to evaluate the factors that correlated with *dang-qui* use based on odds ratios (ORs) and the 95% confidence intervals (CIs). Risks of endometrial cancer were taken into consideration, such as age, diabetes mellitus, hypertension, the cumulative dose of tamoxifen administered (<7,500 mg, 7,500–14,999 mg, 15,000–29,999 mg, ≥30,000 mg), and the cumulative DDD of tamoxifen administered (<180 DDD, 180–599 DDD, 600–1,199 DDD, ≥1,200 DDD.

Among 31,938 tamoxifen-treated BC survivors from January 1, 1998, to December 31, 2008 in Taiwan, we identified 157 patients who were newly diagnosed with subsequent endometrial cancer from the first date of filling a prescription for tamoxifen to the diagnosis of endometrial cancer, to allow at least 1 years to give sufficient time for BC survivors to accumulate sufficient doses of tamoxifen to induce subsequent endometrial cancer. Eligible period was considered the period either between either before the diagnosis of newly developed endometrial cancer, the death of patient, withdrawal from Taiwan's Health Insurance program, or the end of the study and a BC survivor's first tamoxifen prescription. Cox proportional hazard regression was performed to calculate the adjusted hazard ratio (aHR) for subsequent endometrial cancer among tamoxifen-treated BC survivors who used a CHP containing *dang-qui* and among those who did not use the Chinese herb. To minimise the potential confounding by either oral-contraceptive use or a different predisposition to develop second cancers in the endometrium, we conducted another Cox regression to calculate the adjusted hazard ratio (aHR) for subsequent endometrial cancer among tamoxifen-treated BC survivors aged 55–79 years who used a CHP containing *dang-qui* and among those who did not use the Chinese herb. To explore the potential attrition bias by patient non-compliance (death or withdrawal before the end of the study), we further conducted another Cox regression to calculate the adjusted hazard ratios for subsequent endometrial cancer among the per-prorocal tamoxifen-treated BC survivors (excluding those who leaving Taiwan or died from any diseases before either the diagnosis of endometrial cancer or the end of the study from the intention-to-treat tamoxifen-treated BC survivors) who used a CHP containing dang-qui and among those who did not use the Chinese herb. An estimate with a 95% CI that did not contain the number 1 was considered statistically significant. The SAS statistical software package version 9.3 (SAS Institute, Cary, NC, USA) was used for data management and analysis.

## Results

The database of outpatient claims from 1998 to 2008 contained information on 31,938 tamoxifen-treated BC survivors. Among them, 14,934 (46.7%) breast cancer survivors used *dang-qui* during the study period. Table 1 summarizes the characteristics of the study population, the number of incidents of endometrial cancer, the cumulative doses of tamoxifen, and cancer treatment modalities. The mean age of the non TCM users was higher than that of the *dang-qui* users. There were more non TCM users than *dang-qui* users with no income and more non TCM users than *dang-qui* users who had received tamoxifen for less than 7,500 mg. Within this population, we identified 157 patients who were diagnosed with subsequent endometrial cancer after tamoxifen treatment (*dang-qui* users, n = 77; non TCM users, n = 80) over the 11-year study period (1998–2008).

**Table 1:** Demographic characteristics and results of multiple logistic regression showing the adjusted odds ratio (aOR) and 95% CI (confidence interval) of tamoxifen-treated breast cancer survivors from the National Health Insurance catastrophic illnesses registry of Taiwan between 1998 and 2008.

Characteristics	Tamoxifen users who had used dang-qui (%)	Tamoxifen users without TCM usage (%)	aOR^a^ (95% CI^b^)
No. of cases	14,934	17,004	
No. of endometrial cancer	77	80	
Age at diagnosis of breast cancer			
Average	50.0±10.7	52.4±11.8	
20∼29 yrs	205(1.4)	194(1.1)	1.16(0.94–1.42)
30∼39 yrs	2,058(13.8)	1,982(11.7)	1.04(0.96–1.12)
40∼49 yrs	5,756(38.5)	5,701(33.5)	1
50∼59 yrs	3,976(26.6)	4,462(26.2)	0.93(0.87–0.98)
60∼69 yrs	2,123(14.2)	2,901(17.1)	0.76(0.70–0.81)
70∼79 yrs	817(5.5)	1,764(10.4)	0.51(0.46–0.56)
Insured salaries ($NT/month)			
0+	2,565(17.2)	3,627(21.3)	1
1–19999	7,969(53.4)	8,848(52.0)	1.14(1.07–1.21)
20000–39999	2,940(19.7)	3,079(18.1)	1.21(1.13–1.31)
≥40000	1,460(9.8)	1,450(8.5)	1.27(1.16–1.40)
Insured region			
Northern Taiwan	7,428(49.7)	9,753(57.4)	1
Central Taiwan	3,005(20.1)	2,370(13.9)	1.80(1.69–1.92)
Southern Taiwan	3,997(26.8)	4,316(25.4)	1.25(1.19–1.32)
Eastern Taiwan	330(2.2)	354(2.1)	1.27(1.09–1.49)
Outlying islands	174(1.2)	211(1.2)	1.09(0.88–1.34)
Cumulative doses of tamoxifen			
<7,500 mg	4,270(28.6)	8,090(47.6)	1
7,500–14,999 mg	2,849(19.1)	3,279(19.3)	1.60(1.50–1.70)
15,000–29,999 mg	4,428(29.6)	3,357(19.7)	1.82(1.52–2.18)
≥30,000 mg	3,387(22.7)	2,278(13.4)	2.16(1.79–2.62)
Cancer treatment modalities			
Tamoxifen only	208(1.4)	386(2.3)	1
Tamoxifen and chemotherapy	336(2.2)	777(4.6)	0.73(0.59–0.91)
Tamoxifen plus surgery	3,869(25.9)	4,558(26.8)	1.12(0.93–1.34)
Tamoxifen plus surgery, plus chemotherapy	10,521(70.4)	11,283(66.4)	1.13(0.94–1.35)

a , OR refers to odds ratio;

b , CI refers to confidence interval.

The adjusted odds ratios (aORs) and 95% confidence intervals (95% CIs) obtained by multiple logistic regression are summarized in Table 1. Compared with the age group 40–49 years (aOR = 1.00), there were significant differences in ages between the *dang-qui* users and the non TCM users aged 50 years and over, who were significantly less likely to use Chinese herbs. tamoxifen-treated BC survivors with a higher income (1–19,999: OR = 1.14; 95% CI: 1.07–1.21, 20,000–39,999: OR = 1.21; 95% CI: 1.13–1.31, and>40,000: OR = 1.27; 95% CI: 1.16–1.40) were more likely to be *dang-qui* users than were the patients with no income. Tamoxifen-treated BC survivors with a higher doses of tamixofen use were associated with a higher use of *dang-qui* in the study population.

An analysis of the major disease categories for the 14,934 *dang-qui* users (Table 2) showed that breast cancer was the most common reason for using a CHP containing *dang-qui* (30.7%, visits = 43,477), followed by “symptoms, signs, and ill-defined conditions” (20.2%, visits  = 28,615), and “diseases of the musculoskeletal system and connective tissue” (11.1%, visits  = 15,703). Table 3 summarizes the different magnitudes of endometrial cancer risks between *dang-qui* users and non TCM users after adjusting for age, diabetes mellitus and hypertension, demographic variables, and cumulative tamoxifen dose. We observed a decreased hazard ratio between the intake of *dang-qui* and endometrial cancer, which suggests that *dang-qui* is unlikely to be the agent responsible for carcinogenicity among the tamoxifen-treated BC survivors. Compared to non TCM users, the adjusted HR for the development of endometrial cancer was decreased among *dang-qui* users by 0.40-fold (95% CI 0.21–0.74) for the cumulative dose of tamoxifen administered less than 7,500 mg group, and 0.32–fold (95% CI 0.11–0.91) for less than 180 DDD group. However, the hazard ratio of subsequent endometrial cancer was slightly increased among *dang-qui* users aged 55–79 years than that of tamoxifen-treated BC survivors with the same age group who did not use Chinese herbs, abet no significantly, if the BC survivors who had received a cumulative tamoxifen doses more than 30.000 mg (HR: 1.61, 95% CI: 0.42–6.14) or 600 DDD (600–1,199 DDD group: HR: 1.22, 95% CI: 0.52–2.87) were examined, as shown in Table 4. 4,361 tamoxifen-treated BC survivors leaving Taiwan or died from any diseases before either the diagnosis of endometrial cancer or the end of the study were found. Among them, 1,768 were *dang-qui* users, whereas the remaining 2,593 were TCM non users. The final observed results in per-protocal population consistent with the results in intention-to-treat population.

**Table 2:** Frequency distribution of visits associated with the prescription of traditional Chinese medicine (TCM) with dang-qui by major disease categories (according to the ICD-9 codes) among tamoxifen-treated breast cancer survivors from the National Health Insurance catastrophic illnesses registry of Taiwan between 1998 and 2008.

Major disease category	ICD-9-CM code range	Visit	%
Neoplasms	140–239	46,008	32.4
(Including breast cancer)	174	(43,477)	(30.7)
Symptoms, signs, and ill-defined conditions	780–799	28,615	20.2
Diseases of the musculoskeletal system and connective tissue	710–739	15,703	11.1
Diseases of the genitourinary system	580–629	10,914	7.7
Diseases of the digestive system	520–579	10,548	7.4
Diseases of the respiratory system	460–519	9,479	6.7
Injury and poisoning	800–999	4,901	3.5
Diseases of the nervous system and sense organs	320–389	4,167	2.9
Diseases of the skin and subcutaneous tissue	680–709	3,207	2.3
Diseases of the circulatory system	390–459	2,810	2.0
Others		5,416	3.8
Total		141,762	100

**Table 3:** Number (No.) of new cases, population-at-risk, estimated hazard ratios (HRs) and 95% confidence intervals (CIs) for endometrial cancer that were estimated using the multivariate Cox regression model based on data from the National Health Insurance catastrophic illnesses registry of Taiwan among all tamoxifen-treated breast cancer women 20–79 years followed from 1998 to 2008.

Presence of endometrial cancer during the follow-up period	tamoxifen users using Chinese medicine containing *dang-qui* one or more times. (No. of cases/person-years)	tamoxifen users who had never used Chinese medicine. (No. of cases/person-years)	tamoxifen users who had used Chinese medicine containing *dang-qui* versus tamoxifen users who had never used Chinese medicine. (aHR* (95%CI))
**Total tamoxifen user**	77/78,459	80/51,675	0.61(0.44–0.84)
Incidence rate+	98.1	154.8	
Cumulative tamoxifen dose			
<7,500 mg	15/16,371	31/12,824	0.40(0.21–0.74)
7,500–14,999 mg	18/12,195	18/8,919	0.77(0.39–1.52)
15,000–29,999 mg	30/25,275	21/15,089	0.85(0.48–1.50)
≥30,000 mg	14/24,619	10/14,844	0.72(0.32–1.64)
Cumulative tamoxifen dose			
<180 DDD	5/9,571	14/7,381	0.32(0.11–0.91)
180–599 DDD	17/14,062	31/10,900	0.43(0.23–0.79)
600–1,199 DDD	30/19,851	18/12,751	1.05(0.58–1.90)
≥1,200 DDD	25/34,975	17/20,643	0.79(0.42–1.49)

*adjusted for age at breast cancer diagnosed, insured salary, insured region, cumulative tamoxifen dose, and cancer treatment modalities.

+ per 100,000.

**Table 4:** Number (No.) of new cases, population-at-risk, estimated hazard ratios (HR) and 95% confidence intervals (CI) for endometrial cancer estimated using the multivariate Cox regression model using the National Health Insurance catastrophic illnesses registry of Taiwan among all tamoxifen-treated breast cancer women 55–79 years followed from 1998 to 2008.

Presence of endometrial cancer during the follow-up period	tamoxifen users who had used Chinese medicine containing *dang-qui* one or more times. (No. of cases/person-years)	tamoxifen users who had never used Chinese medicine. (No. of cases/person-years)	tamoxifen users who had used Chinese medicine containing *dang-qui* versus tamoxifen users who had never used Chinese medicine. (aHR* (95%CI))
**Total tamoxifen user**	37/24,283	38/20,006	0.74(0.46–1.17)
Incidence rate+	152.4	198.9	
Cumulative tamoxifen dose			
<7,500 mg	7/5,377	16/5,454	0.46(0.18–1.13)
7,500–14,999 mg	6/3,536	8/3,443	0.74(0.25–2.19)
15,000–29,999 mg	15/7,398	11/5,441	0.94(0.42–2.08)
≥30,000 mg	9/7,972	3/5,667	1.61(0.42–6.14)
Cumulative tamoxifen dose			
<180 DDD	3/3,072	7/3,278	0.41(0.10–1.68)
180–599 DDD	6/4,415	13/4,238	0.54(0.20–1.47)
600–1,199 DDD	14/5,728	9/4,729	1.22(0.52–2.87)
≥1,200 DDD	14/11,069	9/7,761	0.95(0.40–2.23)

*adjusted for age at breast cancer diagnosed, insured salary, insured region, cumulative tamoxifen dose, and cancer treatment modalities.

+ per 100,000.

## Discussion

According to our review of the literature, this study is the first large-scale retrospective study exploring the use of CHP containing *dang-qui* among tamoxifen-treated breast cancer survivors. The main finding of this population-based cohort study was that the use of *dang-qui* is common among tamoxifen-treated BC survivors in Taiwan and that such use seems to be associated with certain patient characteristics. TCM is a unique traditional therapy approach for various ailments that has been used in Taiwan for hundreds of years. Such a long period of use may contribute significantly to the high prevalence of *dang-qui* use in tamoxifen-treated BC survivors. The present study includes all patients who were newly diagnosed with breast cancer and were treated with tamoxifen by qualified oncologists between 1998 and 2008 in Taiwan; importantly the rate of insured individuals has been consistently above 96% since 1997, and therefore we can rule out the possibility of selection and recall bias.

One distinguishing feature of the national health care system in Taiwan is the coexistence of modern western medicine and TCM, which includes acupuncture, manipulative therapies as well as CHPs. Breast cancer survivors in Taiwan are free to choose from care offered by oncologists or by TCM clinics; this can lead to a certain level of administered of a CHP containing *dang-qui* during tamoxifen treatment. Although we respect the patients' choice of medical care, we recommend that health care providers need to carefully monitor the potential side effects of *dang-qui* when it is being used alongside or in lieu of tamoxifen. It is worth noting that, despite the fact that there is inadequate data on the clinical safety and add-on effects of *dang-qui* among tamoxifen-treated BC survivors, a large number of TCM practitioners have prescribed CHPs containing *dang-qui*. Clearly, this could possibly result in potential herb-drug interactions and might have an unpredictable effect on tamoxifen's efficacy. Therefore, we suggest that a more critical attitude toward the use of tamoxifen and *dang-qui* in combination is needed among both physicians and breast cancer survivors.

The present findings show that, among tamoxifen-treated BC survivors, those with no income, aged 50 years or above and living in central and southern Taiwan were more likely to be non TCM users, as shown in Table 1. BC survivors who took tamoxifen for the longer period or received optimal surgery plus tamoxifen are more likely to be exposed to *dang-qui*. As shown in Table 2, “breast cancer” was the most common reason for TCM visits that resulted in the prescription of a CHP containing *dang-qui* (30.7%, n = 43,477) and this was followed by “symptoms, signs, and ill-defined conditions” (20.2%, n = 28,615), and then “diseases of musculoskeletal system and connective tissue” (11.1%, n = 15,703). The results indicate that, in addition to breast cancer care, health care providers should pay more attention to the general health conditions of patients who are suffering from either cancer-derived or tamoxifen-induced symptoms and provide proactive recommendations to meet their medical needs.

Our findings demonstrated that 39.8% of all tamoxifen-treated BC survivors in Taiwan had consumed *dang-qui* one or more times during 1998 to 2008. In this context, it is worth noting that *dang-qui* users were exposed to greater cumulative tamoxifen doses than non TCM users over the 11-year study period. Our current estimate of subsequent endometrial cancer cases per million person-years among tamoxifen-treated BC survivors among those who ever consumed *dang-qui* is no higher than those who had received no TCM treatment as shown in Table 3. Although some studies in Western countries have indicated a positive association between endometrial cancer and tamoxifen exposure [7], [8], [22]–[33], there have been few if any human studies exploring the potential increased risk of toxicity when either BC survivors are treated with tamoxifen in Asia population or tamoxifen-treated BC survivors are treated with a CHP containing *dang-qui*. The present findings shown in Table 5 corroborate the results of the National Surgical Adjuvant Breast and Bowel Project, which demonstrated that the hazard rate of endometrial cancer risk among BC survivors was 1.2/1000 patient-years. We also found that BC survivors with tamoxifen use had a 7.5-fold increased risk of endometrial cancer than tamoxifen non-users in Taiwan (Table 5) [34].

**Table 5:** Number of new endometrial cancer cases and estimated hazard ratios (HR), 95% confidence intervals (CI) estimated from Cox regression model among breast cancer survivors with or without tamoxifen treatment stratified by age and followed from 2000 to 2008 a.

	tamoxifen users	tamoxifen non-users	
	(No. of cases/person-years)	(No. of cases/person-years)	HR (95%CI)
Total number	225/188,250	67/237,961	7.50(5.03–11.18)
Incidence rate^b^	119.5	28.2	
Age			
20–24	1/881	0/3,434	-
25–29	2/3,150	2/8,058	8.17(0.63–106.58)
30–34	9/9,731	6/16,633	5.80(1.09–30.78)
35–39	16/20,337	9/28,050	21.03(3.97–111.47)
40–44	36/30,580	6/37,340	21.66(5.28–88.79)
45–49	34/34,235	17/45,347	5.04(2.19–11.59)
50–54	37/24,007	10/33,024	6.45(2.41–17.27)
55–59	22/18,866	7/22,766	7.41(1.91–28.67)
60–64	26/16,775	4/18,859	8.23(2.08–32.49)
65–69	17/11,701	3/11,107	12.54(3.11–50.50)
70–74	14/8,683	1/6,814	10.33(1.07–100.12)
75–79	7/5,287	1/3,927	1.16(0.07–20.46)
80–84	2/2,595	1/1,727	-
85+	2/1,423	0/876	-

a Data from the Taiwan National Health Research Institute Database.

b Rates are average annual per 100,000.

Table 3 shows that, when tamoxifen-treated BC survivors who had never consumed any Chinese medicine were used as the reference group, there was a decreased risk of subsequent endometrial cancer among *dang-qui* consumers after taking the cumulative dose of tamoxifen treatment into consideration. Just as important, among women of reproductive age, tamoxifen has an antiestrogenic effect on the uterus, whereas among postmenopausal women, tamoxifen has an estrogenic effect [35]. In addition, in order to minimize among potential confounding factors that might be associated with either the potential consumption of oral contraceptives or with a different predisposition to develop second cancers in the endometrium, we limited the analysis to women aged 55 to 79 years. Again, there was a decrease in the risk of subsequent endometrial cancer among *dang-qui* consumers found for the lower than 7,500.mg cumulative dose group, or the lower than 180 cumulative DDD group, although not significantly.

Previous study indicated that a synergistic antiproliferative effect was observed when n-butylidenephthalide, which is isolated from a chloroform extract of *dang-qui*, was combined with the chemotherapeutic agent 1,3-bis(2-chloroethyl)-1-nitrosourea through inhibition of the O^6^-methylguanine methyltransferase (MGMT) protein expression [36]. In addition, the mechanism of action of tamoxifen is through inhibition of the estrogen receptor and is able to decrease the level of MGMT protein [37]. It is reasonable to infer that the present result might be due to a synergistic antiproliferative effect when *dang-qui* was combined with tamoxifen via a mechanism involving inhibiting the MGMT protein expression. However, further studies are warranted to clarify safety concerns regarding the potential *dang-qui*-tamoxifen interaction and to investigate the efficacy of concurrent use of tamoxifen and *dang-qui*.

To our limited knowledge, the present data is the first of its kind to report a negative relationship between *dang-qui* consumption and subsequent endometrial cancer after less than 7,500 mg or 600 DDD tamoxifen treatment among BC survivors aged 55–79 years. It is worth noting that among the BC survivors aged 55–79 years who had received more than a cumulative tamoxifen doses greater than 7,500 mg or 600 DDD, risk was, although not statistically significantly, slightly greater among those who ever consumed *dang-qui* than among those who never receive a Chinese herb. Therefore, before drawing any conclusions from these findings, further studies among breast cancer survivors who receiving long-term tamoxifen administration are warranted in which an analysis of endometrial cancer and the add-on effects of *dang-qui* are explored, especially among postmenopausal women.

The present study has some limitations. First, because the identities of the patients were encrypted and thus are not available within the NHI reimbursement database, we were unable to obtain any histopathology reports to verify the diagnoses. However, in Taiwan, oncologists who are involved in the treatment of breast cancer must prescribe in line with the requirements of the NHI and estrogen receptor positive breast cancer needs to be confirmed when claiming reimbursement from the NHI. Second, this study did not include Chinese herbal remedies purchased directly from TCM herbal pharmacies, nor have we included health foods that contain herbs. Thus, the frequency of *dang-qui* utilization might have been underestimated. However, because the NHI system has a comprehensive coverage for TCM prescriptions, the cost of which is generally less than the cost of herbs sold in Taiwan's markets, the likelihood that subjects purchased and consumed large amounts of other herbs outside the NHI database is not high. Third, we were unable to obtain any medical records to identify the reason why tamoxifen-treated BC survivors leaving Taiwan or the cause of death before either the diagnosis of endometrial cancer or the end of the study. Worth of note, the consistent results of endometrial cancer risk between daog-qui consumers and non TCM users in either the intention-to-treat population or per-protocal population were observed, the likelihood of a bias introduced by using compliance is not high. An important drawback of this study is the low number of cases, which hindered stratified analysis by menopausal status at diagnosis, resulting in reducing chance of detecting a true effect.

## Conclusions

It is apparent that our findings have implications for physicians attending to tamoxifen-treated BC survivors. Our results suggest that, when there is co-existence of the conventional medical treatments and TCM, the majority of tamoxifen-treated BC survivors consume herbal therapies containing *dang-qui* with the intention of relieving their breast cancer and tamoxifen-induced symptoms. The negative relationship between *dang-qui* consumption and subsequent endometrial cancer after less than a cumulative dose of 7,500 mg among BC survivors is worthy of note. However, further studies among breast cancer survivors who have received long-term tamoxifen administration are warranted in order to explore further the relationship between endometrial cancer and the add-on effects of *dang-qui*, especially among postmenopausal women.

## References

[1] Bernstein L, Deapen D, Cerhan JR, Schwartz SM, Liff J, et al (1999) Tamoxifen therapy for breast cancer and endometrial cancer risk. J Natl Cancer Inst 91:1654–1662. 10511593 10.1093/jnci/91.19.1654

[2] van Leeuwen FE, Benraadt J, Coebergh JW, Kiemeney LA, Gimbrere CH, et al (1994) Risk of endometrial cancer after tamoxifen treatment of breast cancer. Lancet 343:448–452. 7905955 10.1016/s0140-6736(94)92692-1

[3] Cook LS, Weiss NS, Schwartz SM, White E, McKnight B, et al (1995) Population-based study of tamoxifen therapy and subsequent ovarian, endometrial, and breast cancers. J Natl Cancer Inst 87:1359–1364. 7658496 10.1093/jnci/87.18.1359

[4] Nayfield SG, Karp JE, Ford LG, Dorr FA, Kramer BS (1991) Potential role of tamoxifen in prevention of breast cancer. J Natl Cancer Inst 83:1450–1459. 1920492 10.1093/jnci/83.20.1450

[5] Bush TL, Helzlsouer KJ (1993) Tamoxifen for the primary prevention of breast cancer: a review and critique of the concept and trial. Epidemiol Rev 15:233–243. 8405207 10.1093/oxfordjournals.epirev.a036110

[6] Gelmon K (2000) One step forward or one step back with tamoxifen? Lancet 356:868–869. 11036885 10.1016/S0140-6736(00)02670-2

[7] Curtis RE, Boice JD Jr, Shriner DA, Hankey BF, Fraumeni JF Jr. (1996) Second cancers after adjuvant tamoxifen therapy for breast cancer. J Natl Cancer Inst 88:832–834. 8637050 10.1093/jnci/88.12.832

[8] Early Breast Cancer Trialists' Collaborative Group (1998) Tamoxifen for early breast cancer: an overview of the randomised trials. Lancet 351:1451–1467. 9605801

[9] Bonneterre J, Thurlimann B, Robertson JF, Krzakowski M, Mauriac L, et al (2000) Anastrozole versus tamoxifen as first-line therapy for advanced breast cancer in 668 postmenopausal women: results of the Tamoxifen or Arimidex Randomized Group Efficacy and Tolerability study. J Clin Oncol 18:3748–3757. 11078487 10.1200/JCO.2000.18.22.3748

[10] Reyes-Gibby CC, Wu X, Spitz M, Kurzrock R, Fisch M, et al (2008) Molecular epidemiology, cancer-related symptoms, and cytokines pathway. Lancet Oncol 9:777–785. 18672213 10.1016/S1470-2045(08)70197-9 PMC3390774

[11] Tagliaferri M, Cohen I, Tripathy D (2001) Complementary and alternative medicine in early-stage breast cancer. Semin Oncol 28:121–134. 11254871 10.1016/s0093-7754(01)90049-1

[12] Rakovitch E, Pignol JP, Chartier C, Ezer M, Verma S, et al (2005) Complementary and alternative medicine use is associated with an increased perception of breast cancer risk and death. Breast Cancer Res Treat 90:139–148. 15803360 10.1007/s10549-004-3779-1

[13] Department of Chinese Medicine and Pharmacy, Ministry of Health and Welfare, Executive Yuan, Taiwan (2014) List of 21 most important ancient text in Chinese medicine.

[14] Lai JN, Wu CT, Wang JD (2012) Prescription pattern of chinese herbal products for breast cancer in taiwan: a population-based study. Evid Based Complement Alternat Med 2012:891893. 22685488 10.1155/2012/891893 PMC3368194

[15] Lai JN, Tang JL, Wang JD (2013) Observational Studies on Evaluating the Safety and Adverse Effects of Traditional Chinese Medicine. Evid Based Complement Alternat Med 2013:697893. 24159351 10.1155/2013/697893 PMC3789390

[16] Lee KH, Tsai YT, Lai JN, Lin SK (2013) Concurrent Use of Hypnotic Drugs and Chinese Herbal Medicine Therapies among Taiwanese Adults with Insomnia Symptoms: A Population-Based Study. Evid Based Complement Alternat Med 2013:987862. 24204397 10.1155/2013/987862 PMC3800591

[17] Chen MC, Lai JN, Chen PC, Wang JD (2013) Concurrent Use of Conventional Drugs with Chinese Herbal Products in Taiwan: A Population-based Study. Journal of Traditional and Complementary Medicine 3:256–262. 24716186 10.4103/2225-4110.119734 PMC3925000

[18] Lee YC, Huang YT, Tsai YW, Huang SM, Kuo KN, et al (2010) The impact of universal National Health Insurance on population health: the experience of Taiwan. BMC Health Serv Res 10:225. 20682077 10.1186/1472-6963-10-225 PMC2924329

[19] Department of Chinese Medicine and Pharmacy, Ministry of Health and Welfare, Executive Yuan, Taiwan (2013) List of 100 unified formulae.

[20] Centers for Disease Control and Prevention (2013) International Classification of Diseases, Ninth Revision, Clinical Modification (ICD-9-CM).

[21] Hsieh SC, Lai JN, Lee CF, Hu FC, Tseng WL, et al (2008) The prescribing of Chinese herbal products in Taiwan: a cross-sectional analysis of the national health insurance reimbursement database. Pharmacoepidemiol Drug Saf 17:609–619. 18481335 10.1002/pds.1611

[22] Cuzick J, Powles T, Veronesi U, Forbes J, Edwards R, et al (2003) Overview of the main outcomes in breast-cancer prevention trials. Lancet 361:296–300. 12559863 10.1016/S0140-6736(03)12342-2

[23] Swerdlow AJ, Jones ME. British Tamoxifen Second Cancer Study Group (2005) Tamoxifen treatment for breast cancer and risk of endometrial cancer: a case-control study. J Natl Cancer Inst 97:375–384. 15741574 10.1093/jnci/dji057

[24] Hardell L (1988) Pelvic irradiation and tamoxifen as risk factors for carcinoma of corpus uteri. Lancet 2:1432. 10.1016/s0140-6736(88)90629-0 2904564

[25] Fornander T, Rutqvist LE, Cedermark B, Glas U, Mattsson A, et al (1989) Adjuvant tamoxifen in early breast cancer: occurrence of new primary cancers. Lancet 1:117–120. 2563046 10.1016/s0140-6736(89)91141-0

[26] Andersson M, Storm HH, Mouridsen HT (1991) Incidence of new primary cancers after adjuvant tamoxifen therapy and radiotherapy for early breast cancer. J Natl Cancer Inst 83:1013–1017. 2072407 10.1093/jnci/83.14.1013

[27] Fisher B, Costantino JP, Redmond CK, Fisher ER, Wickerham DL, et al (1994) Endometrial cancer in tamoxifen-treated breast cancer patients: findings from the National Surgical Adjuvant Breast and Bowel Project (NSABP) B-14. J Natl Cancer Inst 86:527–537. 8133536 10.1093/jnci/86.7.527

[28] Rutqvist LE, Johansson H, Signomklao T, Johansson U, Fornander T, et al (1995) Adjuvant tamoxifen therapy for early stage breast cancer and second primary malignancies. Stockholm Breast Cancer Study Group. J Natl Cancer Inst 87:645–651. 7752269 10.1093/jnci/87.9.645

[29] Sasco AJ (1996) Tamoxifen and menopausal status: risks and benefits. Lancet 347:761. 10.1016/s0140-6736(96)90111-7 8602018

[30] Mignotte H, Lasset C, Bonadona V, Lesur A, Luporsi E, et al (1998) Iatrogenic risks of endometrial carcinoma after treatment for breast cancer in a large French case-control study. Federation Nationale des Centres de Lutte Contre le Cancer (FNCLCC). Int J Cancer 76:325–330. 9579567 10.1002/(sici)1097-0215(19980504)76:3<325::aid-ijc7>3.0.co;2-x

[31] Bergman L, Beelen ML, Gallee MP, Hollema H, Benraadt J, et al (2000) Risk and prognosis of endometrial cancer after tamoxifen for breast cancer. Comprehensive Cancer Centres' ALERT Group. Assessment of Liver and Endometrial cancer Risk following Tamoxifen. Lancet 356:881–887. 11036892 10.1016/s0140-6736(00)02677-5

[32] Rubino C, de Vathaire F, Shamsaldin A, Labbe M, Le MG (2003) Radiation dose, chemotherapy, hormonal treatment and risk of second cancer after breast cancer treatment. Br J Cancer 89:840–846. 12942115 10.1038/sj.bjc.6601138 PMC2394476

[33] Curtis RE, Freedman DM, Sherman ME, Fraumeni JF_cc Jr. (2004) Risk of malignant mixed mullerian tumors after tamoxifen therapy for breast cancer. J Natl Cancer Inst 96:70–74. 14709741 10.1093/jnci/djh007

[34] Love RR (1994) Re: Endometrial cancer in tamoxifen-treated breast cancer patients: findings from the National Surgical Adjuvant Breast and Bowel Project (NSABP) B-14. J Natl Cancer Inst 86:1025–1026. 8007012 10.1093/jnci/86.13.1025

[35] Sunderland MC, Osborne CK (1991) Tamoxifen in premenopausal patients with metastatic breast cancer: a review. J Clin Oncol 9:1283–1297. 2045868 10.1200/JCO.1991.9.7.1283

[36] Yu YL, Yu SL, Su KJ, Wei CW, Jian MH, et al (2010) Extended O6-methylguanine methyltransferase promoter hypermethylation following n-butylidenephthalide combined with 1,3-bis(2-chloroethyl)-1-nitrosourea (BCNU) on inhibition of human hepatocellular carcinoma cell growth. J Agric Food Chem 58:1630–1638. 20043672 10.1021/jf903043r

[37] Kuo CC, Liu JF, Shiah HS, Ma LC, Chang JY (2007) Tamoxifen accelerates proteasomal degradation of O6-methylguanine DNA methyltransferase in human cancer cells. Int J Cancer 121:2293–2300. 17597106 10.1002/ijc.22927

